# Supplementary material for: Regional and temporal variations in COVID-19 cases and deaths in Ethiopia: Lessons learned from the COVID-19 enhanced surveillance and response
Source: PLOS Glob Public Health. 2024 May 23;4(5):e0003175. doi: 10.1371/journal.pgph.0003175 (PMC11115226; doi:10.1371/journal.pgph.0003175)
Supplement: S1 Appendix — (DOCX) [file pgph.0003175.s002.docx]

# **Definition of variables**

**COVID-19 Contact:** A contact is a person who has had any one of the following exposures to a probable or confirmed case:

- Face-to-face contact with a probable or confirmed case within 1 meter and for at least 15 minutes
- Direct physical contact with a probable or confirmed case
- Direct care for a patient with probable or confirmed COVID-19 disease without the use of recommended personal protective equipment or
- Other situations as indicated by local risk assessments.

Exposure must have occurred during the infectious period of the case, and defined as follows:

- Exposure to a symptomatic case: 2 days before and 10 days after symptom onset of the case, plus at least 3 additional days without symptoms or 3 days with improving symptoms (including without fever and without respiratory symptoms), for a minimum of 13 days total after symptom onset.
- Exposure to an asymptomatic case: 2 days before and 10 days after the date on which the sample that led to confirmation was taken.

**COVID-19 Death:** A COVID-19 death is defined for surveillance purposes as a death resulting from a clinically compatible illness in a probable or confirmed COVID-19 case unless there is a clear alternative cause of death that cannot be related to COVID-19 disease (e.g. trauma). There should be no period of complete recovery between the illness and death.

**COVID-19 Related Hospitalization:** Hospitalization is defined as admission as an inpatient for a length of over 12 hours, or overnight. Admission to intensive care for COVID-19 treatment is monitored to capture the severity of COVID-19 disease and its impact on intensive care unit (ICU) capacity.

**New admission to ICU Unit:** An ICU is defined as “an organized system for the provision of care to critically ill patients that provides intensive and specialized medical and nursing care, an enhanced capacity for monitoring, and multiple modalities of physiologic organ support to sustain life during a period of life-threatening organ system insufficiency”.

**COVID-19 Cluster:** A group of symptomatic individuals linked by time, geographic location and common exposures, containing at least one NAAT-confirmed case or at least two epidemiologically linked, symptomatic (meeting clinical criteria of suspect case definition A or B) persons with positive Ag-RDTs performed by a trained operator OR as a self-test (based on ≥97% specificity of test and desired >99.9% probability of at least one positive result being a true positive).
