# Supplementary material for: Regional and temporal variations in COVID-19 cases and deaths in Ethiopia: Lessons learned from the COVID-19 enhanced surveillance and response
Source: PLOS Glob Public Health. 2024 May 23;4(5):e0003175. doi: 10.1371/journal.pgph.0003175 (PMC11115226; doi:10.1371/journal.pgph.0003175)
Supplement: Attachment — Submitted filename: PGPH-D-23-01272_reviewer_comments.pdf [file pgph.0003175.s004.pdf]

PLOS Global Public Health

Regional and temporal variations in COVID-19 cases and deaths in Ethiopia: Lessons learned from the COVID-19 enhanced surveillance and response

--Manuscript Draft--

Manuscript Number:	PGPH-D-23-01272
Article Type:	Research Article
Full Title:	Regional and temporal variations in COVID-19 cases and deaths in Ethiopia: Lessons learned from the COVID-19 enhanced surveillance and response
Short Title:	Regional and temporal variations in COVID-19 cases and deaths in Ethiopia
Corresponding Author:	Nebiyu Dereje, MPH, PhD<br>Addis Ababa University<br>Addis Ababa, ETHIOPIA
Order of Authors:	Gizaw Teka, MD, MPH
	Adane Woldeab, MPH
	Nebiyu Dereje, MPH, PhD
	Frehywot Eshetu, MD, MPH
	Lehageru Gizachew, MD, MPH
	Zelalem Tazu, Masters
	Leuel Lisanwork, MD, MPH
	Eyasu Tigabu, Ph.D.
	Ayele Gebeyehu, MPH
	Adamu Tayachew, Masters
	Mengistu Biru, MPH
	Tsegaye Berkessa, DVM
	Abrham Keraleme, Masters
	Fentahun Bikale, MPH
	Wolde Shure, MPH
	Admikew Agune, MPH
	Bizuwork Haile, MPH
	Beza Addis, MD, MPH
	Muluken Moges, MPH
	Melaku Gonta, MPH
	Aster Hailemariam, Ph.D.
	Laura Binkley, Ph.D.
	Saira Nawaz, Ph.D.
	Shuhua Wang, Ph.D.
	Zelalem Mekuria, Ph.D.
	Ayalew Aklilu, MPH
	Jemal Aliy, MD, MPH
	Sileshi Lulseged, MD, MPH
	Abiy Girmay, Ph.D.

Powered by Editorial Manager® and ProduXion Manager® from Aries Systems Corporation

	Abok Patrick, Ph.D.
	Birhanu Amare, MD, MPH
	Hulemenaw Delelegn, Masters
	Sharon Daves, MPH
	Getnet Yimer
	Ebba Abate, Ph.D.
	Mesfin Wossen, MPH
	Zenebe Melaku, MD, MPH
	Wondwossen Gebreyes, Ph.D.
	Desmond E. E. Williams
	Aschalew Abayneh, MPH
**Keywords:**	COVID-19; Surveillance; Ethiopia; Variation
**Abstract:**	Background<br><br>The COVID-19 pandemic is one of the most devastating public health emergencies of international concern to have occurred in the past century. To ensure a safe, scalable, and sustainable response, it is imperative to understand the burden of disease, epidemiological trends, and responses to activities that have already been implemented. We aimed to analyze how COVID-19 tests, cases, and deaths varied by time and region in the general population and healthcare workers (HCWs) in Ethiopia.<br><br>Methods<br><br>COVID-19 data were captured between October 01, 2021, and September 30, 2022, in 64 systematically selected health facilities throughout Ethiopia. The number of health facilities included in the study was proportionally allocated to the regional states of Ethiopia. Data were captured by standardized tools and formats. Analysis of COVID-19 testing performed, cases detected, and deaths registered by region and time was carried out.<br><br>Results<br><br>We analyzed 215,024 individuals’ data that were captured through COVID-19 surveillance in Ethiopia. Of 215,024 total tests, 18,964 COVID-19 cases (8.8%, 95% CI: 8.7% – 9.0%) were identified and 534 (2.8%, 95% CI: 2.6% – 3.0%) were deceased. The positivity rate ranged from 1% in the Afar region to 15% in the Sidama region. Eight (1.2%, 95% CI: 0.4% – 2.0%) HCWs died out of 664 infected HCWs, of which 81.5% were from Addis Ababa. Three waves of outbreaks were detected during the analysis period, with the highest positivity rate of 35% during the Omicron period and the highest rate of ICU beds and mechanical ventilators (38%) occupied by COVID-19 patients during the Delta period.<br><br>Conclusions<br><br>The temporal and regional variations in COVID-19 cases and deaths underscore the need for concerted efforts to address the disparities in the COVID-19 surveillance and response system. These lessons should be critically considered during the integration of the COVID-19 surveillance system into the routine surveillance system.
**Additional Information:**	
**Question**	**Response**
**Financial Disclosure**	This study was conducted through the analysis of COVID-19 surveillance and response data captured from health facilities included in the U.S. CDC-funded CARES Act project. Its contents are solely the responsibility of the authors and do not necessarily represent the official views of the US Centers for Disease Control and

Enter a financial disclosure statement that describes the sources of funding for the work included in this submission and the role the funder(s) played. This includes grants and any commercial funding of the work or authors.<br><br>This statement will be typeset if the manuscript is accepted for publication.<br><br>*Please review the submission guidelines and the instructions link below for detailed requirements and guidance.*	Prevention (CDC) or the Department of Health and Human Services.
**Competing Interests**<br><br>On behalf of all authors, disclose any competing interests that could be perceived to bias this work.<br><br>This statement will be typeset if the manuscript is accepted for publication.<br><br>*Please review the instructions link below and PLOS Global Public Health's competing interests policy to determine what information must be disclosed at submission.*	None to declare
**Data Availability**<br><br>Before publication, Authors are required to make fully available and without restriction all data underlying their findings. Please see our PLOS Data Policy page for detailed information on this policy.<br><br>A **Data Availability Statement**, detailing where the data can be accessed, is required at first submission. Insert your Data Availability Statement in the box below.	All aggregated COVID-19 surveillance and response data used in this study will be made available in the EPHI data repository after publication. Access to data will be provided following the investigators' approval of a proposal. Request for the data should be directed to the corresponding author. People requesting data will need to sign a data access agreement that will be forwarded upon request and the database will be transferred electronically.

Powered by Editorial Manager® and ProduXion Manager® from Aries Systems Corporation

Please see the data reporting section of our submission guidelines for instructions on what you need to include in your Data Availability Statement.<br><br>This statement will be typeset if the manuscript is accepted for publication.<br><br>*PLOS allows rare exemptions to address legal and ethical concerns. If you have legal or ethical restrictions, please use the box below to detail these in full sentences for the Journal team to consider.*	

Manuscript Click here to access/download;Manuscript;Manuscript.docx

Regional and temporal variations in COVID-19 cases and deaths in Ethiopia: Lessons learned from the COVID-19 enhanced surveillance and response

Gizaw Teka[1, ¶], Adane Woldeab[1,2, ¶], Nebiyu Dereje[3, ¶,*], Frehywot Eshetu[7, ¶], Lehageru Gizachew[2], Zelalem Tazu[2], Leuel Lisanwork[2], Eyasu Tigabu[2], Ayele Gebeyehu[1], Adamu Tayachew[1], Mengistu Biru[1], Tsegaye Berkessa[1], Abrham Keraleme[1], Fentahun Bikale[1], Wolde Shure[1], Admikew Agune[1], Bizuwork Haile[1], Beza Addis[1], Muluken Moges[1], Melaku Gonta[1], Aster Hailemariam[1], Laura Binkley[4], Saira Nawaz[4], Shu-Hua Wang[4,5], Zelalem Mekuria[4], Ayalew Aklilu[3], Jemal Aliy[3], Sileshi Lulseged[3], Abiy Girmay[9], Abok Patrick[9], Berhanu Amare[7], Hulemenaw Delelegn[7], Sharon Daves[7,] Getnet Yimer[8], Ebba Abate[2], Mesfin Wossen[1, &], Zenebe Melaku[3, &], Wondwossen Gebreyes[4,6, &], Desmond E. Williams[7, &], Aschalew Abayneh[1, &]

Affiliation

1. Ethiopian Public Health Institute, Addis Ababa, Ethiopia
2. The Ohio State University Global One Health initiative (GOHi), Addis Ababa, Ethiopia
3. ICAP at Columbia University, Addis Ababa, Ethiopia
4. The Ohio State University Global One Health initiative (GOHi), Columbus, OH, USA
5. The Ohio State University, College of Medicine, Internal Medicine Department, Infectious Disease Division, Columbus, OH, USA
6. The Ohio State University, Department of Veterinary Preventive Medicine, Infectious Diseases Molecular Epidemiology Laboratory, Columbus, OH, USA
7. Division of Global Health Protection (DGHP), US Centers for Disease Control and Prevention (CDC), Addis Ababa, Ethiopia
8. University of Pennsylvania, Perelman School of Medicine, USA
9. World Health Organization (WHO) - Ethiopia

*Corresponding Author

Email: neba.jahovy@gmail.com (ND)

[¶]These authors contributed equally to this work.

[&]These authors also contributed equally to this work.

Abstract

Background: The COVID-19 pandemic is one of the most devastating public health emergencies of international concern to have occurred in the past century. To ensure a safe, scalable, and sustainable response, it is imperative to understand the burden of disease, epidemiological trends, and responses to activities that have already been implemented. We aimed to analyze how COVID-19 tests, cases, and deaths varied by time and region in the general population and healthcare workers (HCWs) in Ethiopia.

Methods: COVID-19 data were captured between October 01, 2021, and September 30, 2022, in 64 systematically selected health facilities throughout Ethiopia. The number of health facilities included in the study was proportionally allocated to the regional states of Ethiopia. Data were captured by standardized tools and formats. Analysis of COVID-19 testing performed, cases detected, and deaths registered by region and time was carried out.

Results: We analyzed 21[illegible]4 individuals' data that were captured through COVID-19 surveillance in Ethiopia. Of 215,024 total tests, 18,964 COVID-19 cases (8.8%, 95% CI: 8.7% – 9.0%) were identified and 534 (2.8%, 95% CI: 2.6% – 3.0%) were deceased. The positivity rate ranged from 1% in the Afar region to 15% in the Sidama region. Eight (1.2%, 95% CI: 0.4% – 2.0%) HCWs died out of 664 infected HCWs, of which 81.5% were from Addis Ababa. Three waves of outbreaks were detected during the analysis period, with the highest positivity rate of 35% during the Omicron period and the highest rate of ICU beds and mechanical ventilators (38%) occupied by COVID-19 patients during the Delta period.

Conclusions: The temporal and regional variations in COVID-19 cases and deaths underscore the need for concerted efforts to address the disparities in the COVID-19 surveillance and response

system. These lessons should be critically considered during the integration of the COVID-19 surveillance system into the routine surveillance system.

Keywords: COVID-19, Surveillance, Ethiopia, Variation

Introduction

The COVID-19 pandemic has been one of the most devastating Public Health Emergencies of International Concern (PHEIC) of this century, with a high burden of cases and deaths globally [1]. The COVID-19 pandemic has tested the resilience of the global public health system and threatened the existing fragile public health systems [2, 3]. To contain the spread of the disease, several global and local initiatives and preventive measures were implemented. Some of these measures were a consequence of the World Health Organization's (WHO) decision to declare COVID-19 as a PHEIC [4] and of the Ethiopian government to declare a State of Emergency, where travel restrictions, school closures, and a ban on public gatherings were implemented.

The WHO recommended COVID-19 surveillance as one of the critical measures necessary to end the pandemic and effectively inform public health responses to limit the spread of the disease and its impacts [5]. It is critical for new cases and clusters of infections to be rapidly identified before widespread transmission occurs [6]. Surveillance for COVID-19 is important to understand long--term epidemiological trends of morbidity and mortality, early warning of changes in epidemiological patterns, the burden of disease on healthcare capacity, circulation of known variants of concern (VOCs), and early detection of new variants of concern. Thus, it was critical to build a resilient public health surveillance system to allow early detection, prevention, and response of COVID-19 in an effective and timely manner [5–7]. To this end, Ethiopia implemented several COVID-19 surveillance and response activities to reduce the spread of the pandemic which included active case detection, contact tracing, care and isolation, and quarantine [8–10].

To strengthen surveillance for COVID-19 and other reportable diseases, a Coronavirus Relief and Economic Security (CARES) Act was enacted by the U.S. government. The U.S. Centers for

Disease Control and Prevention (CDC) funded the Ethiopian National Surveillance Project with CARES funding through a cooperative agreement. The project allowed implementation of different activities through partnership among the Ethiopian Public Health Institute (EPHI), Regional Health Bureaus (RHBs), The Ohio State University Global One Health initiative (GOHi), the ICAP at Columbia University and other partners. Through the project, key interventions to augment the existing COVID-19 surveillance and response system capacity were implemented at both national and regional levels in selected healthcare facilities throughout Ethiopia. Accordingly, COVID-19 surveillance and response data were actively captured from supported healthcare facilities and reported to the EPHI in a systematic and timely manner. As COVID-19 emergency phase ends, Ethiopia is working to integrate its surveillance into the routine public health surveillance system as per the WHO recommendations [11]. Consequently, it is critical to adequately understand the burden of the disease, patterns of outbreaks, and response activities to be sustained and scaled up.

This study report is uniquely comprehensive at the national scale. Only a few regional studies on the clinical and epidemiological profile of COVID-19 cases in Ethiopia have been published [10,12–14]. These studies reported a limited number of cases and lacked information related to the epidemiological and temporal link among the different waves of outbreaks. In addition, prior studies do not adequately correlate surveillance and response efforts carried out during the outbreaks, nor did the studies analyze the burden of the disease among HCWs. To ensure a safe, scalable, and sustainable transition of the surveillance system, it is imperative to understand trends and responses to COVID-19 from the analysis of nationally representative data captured through this collaborative project. Here we describe the burden of COVID-19 (morbidity and mortality),

epidemiological trends, the burden on the healthcare capacity, and response to control spread during the project implementation period.

Materials and Methods

Study Setting, Design, and Selection of Healthcare Facilities

The public health surveillance system in Ethiopia follows the healthcare delivery systems structure with administrative hierarchies within the national health system including the primary healthcare level (health posts, health centers, districts, hospitals), secondary healthcare level (general hospitals), and tertiary healthcare level (specialized comprehensive hospitals). The woreda, zonal, regional, and federal (EPHI) health offices are responsible for coordinating the overall public health surveillance and response systems for each respective level [15]. COVID-19 surveillance was structured in a similar manner, utilizing these healthcare levels [16].

The healthcare facilities (health centers and hospitals) were responsible for capturing COVID-19 cases and daily reporting to higher administrative levels. Our descriptive, cross-sectional study was conducted using the analysis of COVID-19 surveillance and response data captured from 64 of these selected healthcare facilities between October 01, 2021 and September 30, 2022. Among the selected healthcare facilities, 31 were primary level, 21 were secondary level, and 12 were tertiary level healthcare facilities. Selection of facilities was systematically based on reported patient load (high-load facilities were selected), availability of COVID-19 testing service, and access to electricity. The number of healthcare facilities included was proportionally allocated to the regional states of Ethiopia to ensure representation of all geographical areas of the country. However, due to the conflict in Northern Ethiopia (Tigray region), we were unable to include that region in this study. The selected health facilities were supported by the CARES Act project

through provision of designated public health surveillance officers responsible for carrying out COVID-19 surveillance activities to capture data and share reports to the next level in the health system.

Data Collection Tools and Procedures

To ensure the quality of surveillance data and minimize variability, standardized training on COVID-19 surveillance was provided to the public health surveillance officers, continuous mentorship and supportive supervision of officers at reporting facilities were provided regularly (every two months), and completeness and timeliness reports were generated at regional and central levels, with feedback continuously being provided to the reporting facilities.

Surveillance data were captured in healthcare facilities using standardized COVID-19 surveillance tools and case definitions (S1 Appendix) adapted from the national COVID-19 surveillance guideline and the WHO COVID-19 surveillance interim guideline [16,17]. The deployed public health surveillance officers (Master of Public Health degree holders) were responsible for capturing COVID-19 surveillance data from clients, registering cases in the COVID-19 surveillance logbooks, and reporting to higher levels through the DHIS2 system or via e-mailing of Excel spreadsheets. The COVID-19 surveillance tools captured data on suspect case identification and testing status, number of confirmed cases, COVID-19 testing modality, number of admissions and deaths, contacts identified and tested, number of confirmed HCW cases and deaths, number of clusters/outbreaks investigated, and responses provided. Cluster investigation was conducted following WHO standard procedures (S1 Appendix) [18].

Data Management and Analysis

Public health surveillance data were retrieved from different sources (e.g., DHIS2 and Excel spreadsheets) and compiled into one master Excel sheet, cleaned, checked for completeness, and exported to SPSS version 25 software for analysis. Descriptive analysis (frequency and proportions) was carried out and presented in tables and figures while trends of COVID-19 tests, cases, and deaths were presented in line graphs. Calculations for all metrics can be found in Table 1. For analysis, all data were stratified by regional state.

Table 1: Facility-level COVID-19 surveillance metrics and respective calculations used.

Metric	C[illegible]ulation
COVID-19 Positivity Rate	# of confirmed cases/total # tests
Case Fatality Rate	# of confirmed COVID-19-induced deaths/total # confirmed COVID-19 cases
Hospitalization Rate	Total # patients hospitalized for COVID-19/total # confirmed COVID-19 cases
Intensive Care Unit (ICU) Admission Rate	Total # cases admitted to the ICU/total # hospitalized patients
Rate of mechanical ventilator occupied	Total # of mechanical ventilator used / the total # of ventilators in each hospital
Rate of bed occupied	Total # of beds in a facility occupied by COVID-19 patients/ total # of beds in the facility
Surveillance data completeness	Average of the # of h[illegible] facilities that reported in the Epi-week/total # of health facilities expected to report
Surveillance data timeliness	Average of the # of health facilities that reported to EPHI within the prescribed time/total # of health facilities expected to report wi[illegible] the prescribed time (every Tuesday).

Ethical Considerations

This study utilized routine COVID-19 surveillance data; no identifiable, patient-level information was used. Consequently, ethical approval was waived and permission to use the data was obtained from the EPHI.

Role of Funding Source

The funder had no role in the design, processing, analysis, and interpretation of this study.

Results

This study relied on surveillance data from 215,024 COVID-19 suspected cases, contacts, and travelers tested by either the Antigen Detection Rapid Diagnostic Test (Ag RDT) (88.9%) or Real-Time Polymerase Chain Reaction (RT-PCR) (11.1%) from 64 health facilities. The average completeness and timeliness of the data were 98.1% and 97.9%, respectively (Figure 1).

COVID-19 Testing and Case Detection

Out of the 215,024 COVID-19 suspected cases, contacts, and travelers tested, 88.9% were tested by Ag RDT and 11.1% were tested by RT-PCR. However, facilities in the Harari and Benishangul Gumuz regions used only Ag RDT (100%) as a testing modality. The majority of tests were performed at the secondary healthcare level (general hospitals). One-third (33%) of the testing and one-third (33%) of cases were from facilities in Addis Ababa (Figure 2). Among those tested, 18,964 were found to be positive for COVID-19, with a positivity rate of 8.8% (95% CI: 8.7% – 9.0%). The positivity rate ranged from 1% in the Afar region to 15% in the Sidama region (Table 2).

Table 2: Tests, confirmed cases, and positivity rate by regions of Ethiopia

Regions	Total tests	Tests by testing modality		Test by healthcare facility type			Confirmed case (Positivity rate)	Confirmed case (Positivity rate) by facility type		
		Ag-RDT	RT-PCR	Primary level	Secondary level	Tertiary level		Primary level	Secondary level	Tertiary level
Addis Ababa	71094	49684 (69.9%)	21410 (30.1%)	5764 (8.1%)	32132 (45.2%)	33198 (46.7%)	6212 (9%)	1174 (18.9%)	3455 (55.6%)	1583 (25.5%)
Afar	8036	8026 (99.9%)	10 (0.1%)	7609 (94.7%)	427 (5.3%)	0(0%)	77 (1%)	68 (88.3%)	9 (11.7%)	0 (0%)
Amhara	10535	10520 (99.9%)	10 (0.1%)	2713 (25.8%)	675 (6.4%)	7147 (67.8%)	1531 (15%)	326 (21.3%)	37 (2.4%)	1168 (76.3%)
Benishangul Gumuz	9019	9019 (100.0%)	0 (0%)	5489 (60.9%)	3530 (39.1%)	0(0%)	595 (7%)	252 (42.4%)	343 (57.6%)	0 (0%)
Dire Dawa	31603	31587 (99.9%)	10 (0.1%)	15214 (48.1%)	16389 (51.9%)	0(0%)	2293 (7%)	1186 (51.7%)	1107 (48.3%)	0 (0%)
Gambella	6706	6687 (99.7%)	19 (0.3%)	6261 (93.4%)	445 (6.6%)	0(0%)	210 (3%)	176 (83.8%)	34 (16.2%)	0 (0%)
Harari	13093	13093 (100%)	0 (0%)	5951 (45.5%)	3580 (27.3%)	3562 (27.2%)	1181 (9%)	375 (31.8%)	640 (54.2%)	166 (14.1%)
Oromia	34446	34129 (99.1%)	317 (0.9%)	18616 (54.0%)	15830 (46.0%)	0(0%)	4076 (12%)	2584 (63.4%)	1492 (36.6%)	0 (0%)
Sidama	6892	5316 (77.1%)	1576 (22.9%)	0(0%)	5307 (77.0%)	1585 (23.0%)	1077 (16%)	0 (0%)	862 (80.0%)	215 (20.0%)
SNNPR	13549	13410 (99.0%)	139 (1.0%)	0(0%)	4285 (31.6%)	9264 (68.4%)	1305 (10%)	0 (0%)	489 (37.5%)	816 (62.5%)
Somali	10051	9741 (96.9%)	310 (3.1%)	3583 (35.6%)	5393 (53.7%)	1075 (10.7%)	407 (4%)	129 (31.7%)	273 (67.1%)	0 (0%)
Grand Total	215024	191212 (88.9%)	23812 (11.1%)	71200 (33.1%)	87993 (40.9%)	55831 (26.0%)	18964 (8.8%)	6270 (33.1%)	8741 (46.1%)	3953 (20.8%)

Three waves of outbreaks were detected during the analysis period. The first peak occurred during Epi-weeks 39 and 40 of 2021 (October 2021). The second peak occurred from Epi-week 50 of 2021 to Epi-week 2 of 2022 (Dec 12, 2021 – Jan 15, 2022) and sharply declined from Epi-week 2 to Epi-week 4 of 2022 (Jan 15 – 31, 2022). During this second peak period, the positivity rate reached as high as 35%. The curve remained flat until Epi-week 20 of 2022 (May 13, 2022) after which it began to rise and a third wave was detected from Epi-week 24 to Epi-week 26 of 2022 (June 12 – July 02, 2022) after which the curve declined (Figure 3).

COVID-19 Related Hospitalization and Deaths

Among the confirmed COVID-19 cases, 2573 (13.6%) patients were hospitalized. More than two-thirds (67%) of the hospitalizations were recorded during Epi-week 16 of 2022 (April 16, 2022). However, the highest rate of ICU beds and mechanical ventilators (38%) were occupied by COVID-19 patients during Epi-week 40 of 2021 (Figure 4). One out of five (21%, 95% CI: 19.4% - 22.6%) patients hospitalized for COVID-19 (2,573 patients) died (534 patients), with more than a quarter (26.3%) of the deaths recorded at primary Health Care Units (HCUs) (Table 3).

There were 534 deaths reported among the confirmed cases (18,964) with a total CFR of 2.8% (95% CI: 2.6% – 3.0%). The highest CFR (7.4%) was recorded in the Somali region and the highest total number of deaths was reported in Addis Ababa (Figure 5, Table 3).

Table 3: COVID-19 hospitalization and deaths by region and facility type in Ethiopia

Region	Confirmed cases	Death	CFR (%)	Hospitalized cases by facility type ([illegible], %)			Death by facility type (n, %)		
				Primary HCU	Secondary HCU	Tertiary HCU	Primary HCU	Secondary HCU	Tertiary HCU
Addis Ababa	6212	135	2.20	0(0)	401(56.9)	304(43.1)	0(0)	43(10.7)	92(30.3)
Afar	77	2	2.60	4(44.4)	5(55.6)	0(0)	1(25.0)	1(20.0)	0(0)
Amhara	1531	85	5.60	9(2.1)	16(3.8)	397(94.1)	0(0)	4(25.0)	81(20.4)
Benishangul	595	0	0.00	0(0)	8(100)	0(0)	0(0)	0(0)	0(0)
Dire Dawa	2293	31	1.40	0(0)	234(100)	0(0)	0(0)	31(13.2)	0(0)
Gambella	210	0	0.00	1(100.0)	0(0)	0(0)	0(0)	0(0)	0(0)
Harari	1181	33	2.80	2(1.7)	7(5.8)	112(92.6)	0(0)	0(0)	33(29.5)
Oromia	4076	123	3.00	121(27.8)	314(72.2)	0(0)	33(27.3)	90(28.7)	0(0)
Sidama	1077	40	3.70	0(0)	80(34.8)	150(65.2)	0(0)	0(0)	40(26.7)
SNNPR	1305	55	4.20	0(0)	56(18.3)	250(81.7)	0(0)	13(23.2)	42(16.8)
Somali	407	30	7.40	15(14.7)	86(84.3)	1(1.0)	6(40.0)	23(26.7)	1(100)
Grand Total	18964	534	2.80	152(5.9)	1207(46.9)	1214(47.2)	40(26.3)	205(17.0)	289(23.8)

COVID-19 Healthcare Workers (HCWs) Infection and Death

Six hundred sixty-four (3.5%) of the confirmed COVID-19 cases were HCWs, eight of whom died due to COVID-19 (1.2%, 95% CI: 0.37% – 2.03%). Four out of five (81.5%) infected HCWs were from Addis Ababa, and nearly two-thirds (61.6%) of the infected HCWs were from general hospitals (Table 4).

Table 4: Healthcare workers' infection and death by region and facility type

<table>
<tr><th rowspan="2">Region</th><th colspan="4">HCW confirmed cases by facility type</th><th colspan="4">HCW death by facility type (Fatality rate)</th></tr>
<tr><th>Primary HCU</th><th>Secondary HCU</th><th>Tertiary HCU</th><th>Total</th><th>Primary HCU</th><th>Secondary HCU</th><th>Tertiary HCU</th><th>Total</th></tr>
<tr><td>Addis Ababa</td><td>6(1.5%)</td><td>318(81.5%)</td><td>66(16.9%)</td><td>390</td><td rowspan="12">1(12.5%)</td><td rowspan="12">3(37.5%)</td><td rowspan="12">4(50.0%)</td><td rowspan="12">8(100%)</td></tr>
<tr><td>Afar</td><td>3(100%)</td><td>0(0%)</td><td>0(0%)</td><td>3</td></tr>
<tr><td>Amhara</td><td>5(10.0%)</td><td>1(2.0%)</td><td>44(88.0%)</td><td>50</td></tr>
<tr><td>Benishangul</td><td>8(100%)</td><td>0(0%)</td><td>0(0%)</td><td>8</td></tr>
<tr><td>Dire Dawa</td><td>14(32.6%)</td><td>29(67.4%)</td><td>0(0%)</td><td>43</td></tr>
<tr><td>Gambella</td><td>1(100%)</td><td>0(0%)</td><td>0(0%)</td><td>1</td></tr>
<tr><td>Harari</td><td>4(12.1%)</td><td>6(18.2%)</td><td>23(67.7%)</td><td>33</td></tr>
<tr><td>Oromia</td><td>42(53.8%)</td><td>36(46.2%)</td><td>0(0%)</td><td>78</td></tr>
<tr><td>Sidama</td><td>0(0%)</td><td>7(33.3%)</td><td>14(66.7%)</td><td>21</td></tr>
<tr><td>SNNPR</td><td>0(0%)</td><td>2(9.1%)</td><td>20(90.9%)</td><td>22</td></tr>
<tr><td>Somali</td><td>5(33.3%)</td><td>10(66.7%)</td><td>0(0%)</td><td>15</td></tr>
<tr><td>Grand Total</td><td>88(13.3%)</td><td>409(61.6%)</td><td>167(25.2%)</td><td>664</td></tr>
</table>

COVID-19 Response

A total of 12,813 contacts were identified through contact tracing measures of which 9,323 (72.8%) were tested and 1,095 (11.7%) [illegible]re found to be positive for COVID-19. More than 200 clusters of outbreaks were identified and investigated across all regions of Ethiopia, with the greatest number of clusters being investigate[illegible] the Benishangul Gumuz Region (Table 5).

Table 5: COVID- 19 contacts tracing, testing, and cluster investigation

Region	Contact tracing and test result				Number of clusters investigated
	Identified	Tested	% Tested	Positive cases (PR)	
Addis Ababa	3315	2147	64.8	394(18.4%)	25
Afar	258	229	88.8	40(17.5%)	4
Amhara	90	56	62.2	17(30.4%)	20
Benishangul Gumuz	577	505	87.5	104(20.6%)	56
Dire Dawa	4584	3629	79.2	242(6.7%)	31
Gambella	145	97	66.9	12(12.4%)	4
Harari	564	197	34.9	45(22.8%)	17
Oromia	1520	1517	99.8	144(9.5%)	0
Sidama	268	42	15.7	10(23.8%)	0
SNNPR	895	394	44.0	47(11.9%)	15
Somali	597	510	85.4	40(7.8%)	32
Grand Total	12813	9323	72.8	1095(11.7%)	204

Discussion

The principal findings of this study indicate the COVID-19 case and death tolls, positivity rate, and CFR were substantial among the study population as a whole and high among HCWs. COVID-19 has had a tremendous impact on the overall health, social, psychological, and economic well-being of the Ethiopian population. The resiliency and capacity of the public health system were also highly threatened. Moreover, the findings revealed regional variations in COVID-19 testing, testing algorithms, case detection, hospitalization, contact tracing, and cluster investigations. It is important to note the regional disparities in COVID-19 surveillance and response activities could pose challenges to the efforts to control the pandemic. Sustainable and effective integration of the surveillance system into the routine public health surveillance system should consider addressing these regional variations by properly assessing and identifying the gaps.

The completeness and timeliness of the COVID-19 surveillance data in our study surpassed the national and WHO standards [16,17]. This was achieved by the implementation of several measures. First, designated surveillance officers in the selected healthcare facilities had the primary responsibility to carry out COVID-19 surveillance activities. Second, monitoring, supportive supervision, and mentorship activities were robust. Third, the political commitment was high and fourth, electronic DHIS2-based data capturing for COVID-19 was utilized. These implementation lessons need to be scaled up to ensure a sustainable integration of the COVID-19 surveillance system.

Referencing the WHO variant tracking data, we suspect the three waves of COVID-19 outbreaks identified during the one-year period of surveillance data occurred due to changes in the COVID-19 variants in circulation. The first wave (October 2021) was attributed to the Delta variant, which was characterized by higher severity (hospitalization), but a relatively lower positivity rate. This

was when the highest death rate, ICU admission, and mechanical ventilator usage were recorded. The second wave (December 2021) was attributed to the Omicron variant, which was characterized by a higher positivity rate, but lower severity. During this wave, more than one out of three individuals tested were found to be positive for COVID-19. Omicron cases were less symptomatic, resulted in fewer hospital admissions for those who were unvaccinated and for those who were already immunized after the booster dose and were associated with quicker recovery [19,20]. The third wave (January 2022) was attributed to the Omicron subvariant, which had similar characteristics to the Omicron variant in terms of positivity and severity [19].

The overall positivity rate in our study was relatively lower than study reports from other countries. However, there was regional variation in the positivity rate. For instance, consistent with other studies,[13,14] nearly one-third of the tested patients in Addis Ababa (the capital city) was found to be positive for COVID-19, while only 1% and 2% positivity were reported in the Afar and Somali regions, respectively. These findings could be due to facilities are close to the point of entry (PoE) in these two regions, where the majority of the tested clients are border-crossi[illegible] clients. Moreover, the lower positivity rates in the regions might be due to the warm weather of the regions, as consistent findings were reported from a study in Pakistan [21].

The COVID-19 CFR among all confirmed cases is higher in our study than the national CFR (2.8 compared to 2.0%) for the same period. This variation could be explained by the type of surveillance we employed ─ health facility-based versus the national surveillance system, which includes facility-based, community-based, point-of-entry, and mortality surveillance types [16]. It is likely symptomatic and possibly severe COVID-19 cases with comorbidities were captured by facility-based surveillance.

Similar to other countries,[22,23] a substantial number of HCWs were found to be infected and died of COVID-19 in Ethiopia. This has several implications for clinical and public health practices. As the ratio of HCWs to the population in Ethiopia is low, their infection and death substantially affected the capacity of the healthcare system to respond to the pandemic and other diseases. HCWs are part of the basic elements of health systems and their contribution is critical to achieving global health security and universal health coverage [24]. Moreover, the infection and death of HCWs may indicate poor infection prevention and control (IPC) strategies and practices, including limited availability and appropriate use of personal protective equipment (PPE) [25-28]. Therefore, it is critical for the country to adapt and execute critical precautionary measures to reduce HCW infection and death and ensure a safe and sustainable COVID-19 surveillance system is implemented.

This study analyzed data captured from a well-monitored, collaborative COVID-19 surveillance project covering all regions of Ethiopia. The analysis included data captured over a full year period including regional variation in the number (proportion) of tests, cases, deaths, and HCW-associated infections and deaths to fully understand trends and justify identified variations. However, this study has some limitations. Results were not able to capture more fine-scale variation because the study relied on aggregate surveillance data as opposed to individual, patient-level data. COVID-19 surveillance data were not able to be captured in the Tigray region and some parts of the Amhara and Afar regions due to conflict which may limit the generalization of our findings to the country as a whole. Further, the surveillance system may not adequately capture COVID-19 cases with asymptomatic or mild clinical course at the community level.

Conclusions

The temporal and regional variations in COVID-19 cases and deaths in the population and among HCWs underscore the need for concerted efforts to address the disparities in the COVID-19 surveillance and response system and the provision of adequate PPE for the HCWs. These lessons should be critically considered during the integration of the COVID-19 surveillance system into the routine surveillance system to monitor its trends, detect variants of concern and other respiratory diseases, and sustain gains made during the acute phase of the COVID-19 response.

Contributors

GT, AW, ND, FE, LG, ZT conceived and designed the study, collected and analyzed the data, produced the visualizations, and drafted the original manuscript. ET, AG, AT, MB, TB contributed to the study design and data analysis. AK, FB, WS, AA, BH, BA and MM contributed to the monitoring and supervision of the project. MG, AH, LB, SN, SHW, ZM, AAk contributed to the conception, data processing, and analysis. JA, SL, AG, AP, BA, HD, SD, GY contributed with critical input and revision to the manuscript. EA, MW, ZM, WG, DEW, AsA contributed to the conception, design, and monitoring of the study and critically reviewed the manuscript. All authors had access to all the data, contributed to the interpretation of the data, and revised, edited, and approved the final version of the manuscript before submission. All authors had final responsibility for the decision to submit for publication.

Acknowledgments

The authors would like to acknowledge the U.S. CDC for funding this project, the EPHI, RHBs, OSU GOHi, and ICAP at Columbia University for the technical and financial support. We are also

grateful to the surveillance officers deployed in the healthcare facilities and captured the data analyzed in this study.

Declaration of Interests

We declare no competing interests.

Data Sharing

All aggregated COVID-19 surveillance and response data used in this study will be made available in the EPHI data repository after publication. Access to data will be provided following the investigators' approval of a proposal. Request for the data should be directed to the corresponding author. People requesting data will need to sign a data access agreement that will be forwarded upon request and the database will be transferred electronically.

Funding

This study was conducted through the analysis of COVID-19 surveillance and response data captured from health facilities included in the U.S. CDC-funded CARES Act project. Its contents are solely the responsibility of the authors and do not necessarily represent the official views of the US Centers for Disease Control and Prevention (CDC) or the Department of Health and Human Services.

References

[1] Worldometer. Coronavirus Live Updates. https://www.worldometers.info/coronavirus/; 2022 [accessed 25 October 2022].

[2] Hiscott J, Alexandridi M, Muscolini M, et al. The global impact of the coronavirus pandemic. Cytokine Growth Factor Rev. 2020;53:1-9. doi:10.1016/j.cytogfr.2020.05.010

[3] Khetrapal S, Bhatia R. Impact of COVID-19 pandemic on health system & Sustainable Development Goal 3. Indian Journal of Medical Research. 2020;151(5):395. doi:10.4103/ijmr.IJMR_1920_20

[4] Cucinotta D., Vanelli M. WHO Declares COVID-19 a Pandemic. Acta Biomed. 2020;19:91(1).

[5] World Health Organization. Public Health Surveillance for COVID-19: Interim Guidance; https://www.who.int/publications/i/item/WHO-2019-nCoV-SurveillanceGuidance-2022.2; [Accessed 25 October 2022]

[6] Wetterhall SF, Pappaioanou M, Thacker SB, Eaker E, Churchill RE, Centers for Disease Control (CDC). The role of public health surveillance: information for effective action in public health. MMWR Morb Mortal Wkly Rep. 1992;41 Suppl:207-218.

[7] Anema A, Kluberg S, Wilson K, et al. Digital surveillance for enhanced detection and response to outbreaks. Lancet Infect Dis. 2014;14(11):1035-1037. doi:10.1016/S1473-3099(14)70953-3

[8] Lanyero B, Edea ZA, Musa EO, et al. Readiness and early response to COVID-19: achievements, challenges and lessons learnt in Ethiopia. BMJ Glob Health. 2021;6(6):e005581. doi:10.1136/bmjgh-2021-005581

[9] Shigute Z, Mebratie AD, Alemu G, Bedi A. Containing the spread of COVID-19 in Ethiopia. J Glob Health. 2020;10(1). doi:10.7189/jogh.10.010369

[10] Tamiru A, Regassa B, Alemu T, Begna Z. The performance of COVID-19 Surveillance System as timely containment strategy in Western Oromia, Ethiopia. BMC Public Health. 2021;21(1):2297. doi:10.1186/s12889-021-12380-6

[11] World Health Organization. End-to-End Integration of SARS-CoV-2 and Influenza Sentinel Surveillance: Revised Interim Guidance.; 2022.

[12] Watare SH, Alemu MA, Tayachew A, et al. An investigation of a hundred COVID-19 cases and close contacts in Ethiopia, May to June, 2020: A prospective case-ascertained study. PLoS One. 2022;17(10):e0275596. doi:10.1371/journal.pone.0275596

[13] Leulseged TW, Hassen IS, Maru EH, et al. COVID-19 in hospitalized Ethiopian children: characteristics and outcome profile. Pan African Medical Journal. 2022;41. doi:10.11604/pamj.2022.41.16.29414

[14] Alene KA, Gelaw YA, Fetene DM, et al. COVID-19 in Ethiopia: a geospatial analysis of vulnerability to infection, case severity and death. BMJ Open. 2021;11(2):e044606. doi:10.1136/bmjopen-2020-044606

[15] Federal Ministry of Health. Health Sector Transformation Plan II; 2021; https://e-library.moh.gov.et/library/wp-content/uploads/2021/07/HSTP-II.pdf; [accessed 26 October 2022].

[16] Federal Ministry of Health. National Comprehensive COVID-19 Clinical Management Handbook for Ethiopia; 2020; https://e-library.moh.gov.et/library/wp-

content/uploads/2021/07/National-Comprehensive-COVID-19-Clinical-Management-Handbook-Second-Edition.pdf; [accessed 26 October 2022].

[17] World Health Organization. Guidance for Surveillance of SARS-CoV-2 Variants: Interim Guidance; 2021; https://www.who.int/publications/i/item/WHO_2019-nCoV_surveillance_variants; [accessed 26 October 2022].

[18] World Health Organization. Considerations in the Investigation of Cases and Clusters of COVID-19; 2020; https://www.who.int/publications/i/item/considerations-in-the-investigation-of-cases-and-clusters-of-covid-19; [accessed 26 October 2022].

[19] World Health Organization. Tracking SARS-CoV-2 variants; https://www.who.int/activities/tracking-SARS-CoV-2-variants/; [accessed 26 October 2022].

[20] Consolazio D, Murtas R, Tunesi S, et al. A Comparison Between Omicron and Earlier COVID-19 Variants' Disease Severity in the Milan Area, Italy. Frontiers in Epidemiology. 2022;2. doi:10.3389/fepid.2022.891162

[21] Habib G, Shah Zeb Khan M, Gul H, Hayat A, Ur Rehman M. A persistent high ambient temperature waned the community spread of severe acute respiratory syndrome coronavirus-2 in Pakistan. New Microbes New Infect. 2022;45:100961. doi:10.1016/j.nmni.2022.100961

[22] Bandyopadhyay S, Baticulon RE, Kadhum M, et al. Infection and mortality of healthcare workers worldwide from COVID-19: a systematic review. BMJ Glob Health. 2020;5(12):e003097. doi:10.1136/bmjgh-2020-003097

[23] Mehta S, Machado F, Kwizera A, et al. COVID-19: a heavy toll on health-care workers. Lancet Respir Med. 2021;9(3):226-228. doi:10.1016/S2213-2600(21)00068-0

[24] World Health Organization. The Impact of COVID-19 on Health and Care Workers: A Closer Look at Deaths; 2021; https://apps.who.int/iris/handle/10665/345300; [Accessed 28 October 2022].

[25] Ashinyo ME, Dubik SD, Duti V, et al. Infection prevention and control compliance among exposed healthcare workers in COVID-19 treatment centers in Ghana: A descriptive cross-sectional study. PLoS One. 2021;16(3):e0248282. doi:10.1371/journal.pone.0248282

[26] Park SH. Personal Protective Equipment for Healthcare Workers during the COVID-19 Pandemic. Infect Chemother. 2020;52(2):165. doi:10.3947/ic.2020.52.2.165

[27] Galanis P, Vraka I, Fragkou D, Bilali A, Kaitelidou D. Impact of personal protective equipment use on health care workers' physical health during the COVID-19 pandemic: A systematic review and meta-analysis. Am J Infect Control. 2021;49(10):1305-1315. doi:10.1016/j.ajic.2021.04.084

[28] Tian Z, Stedman M, Whyte M, Anderson SG, Thomson G, Heald A. Personal protective equipment (PPE) and infection among healthcare workers – What is the evidence? Int J Clin Pract. 2020;74(11). doi:10.1111/ijcp.13617

 Click here to access/download;Figure;Figure 1.tiff

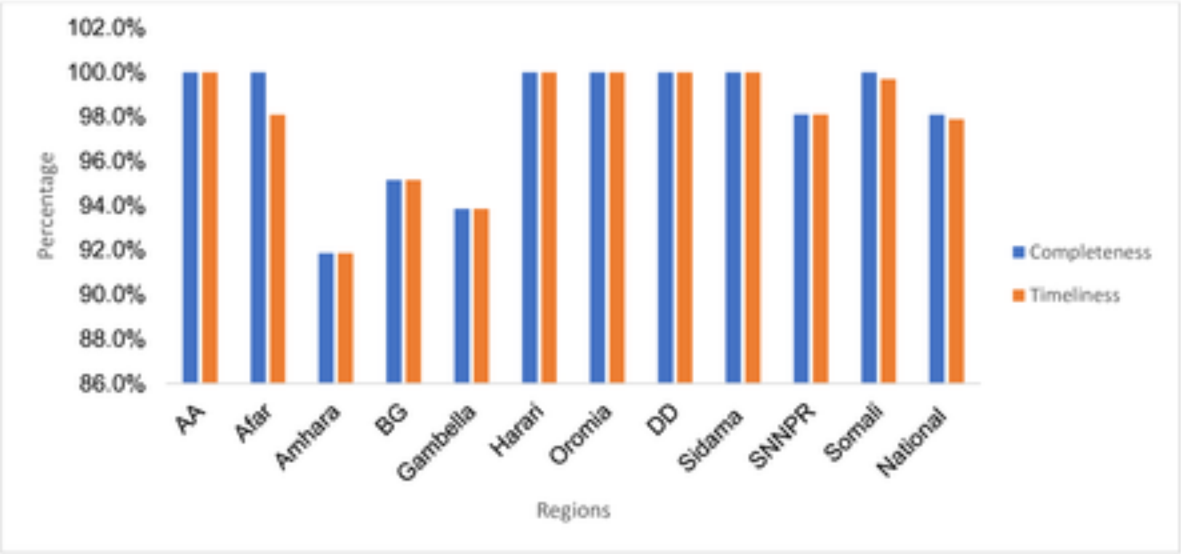

Figure 2

Click here to access/download;Figure;Figure 2.tiff

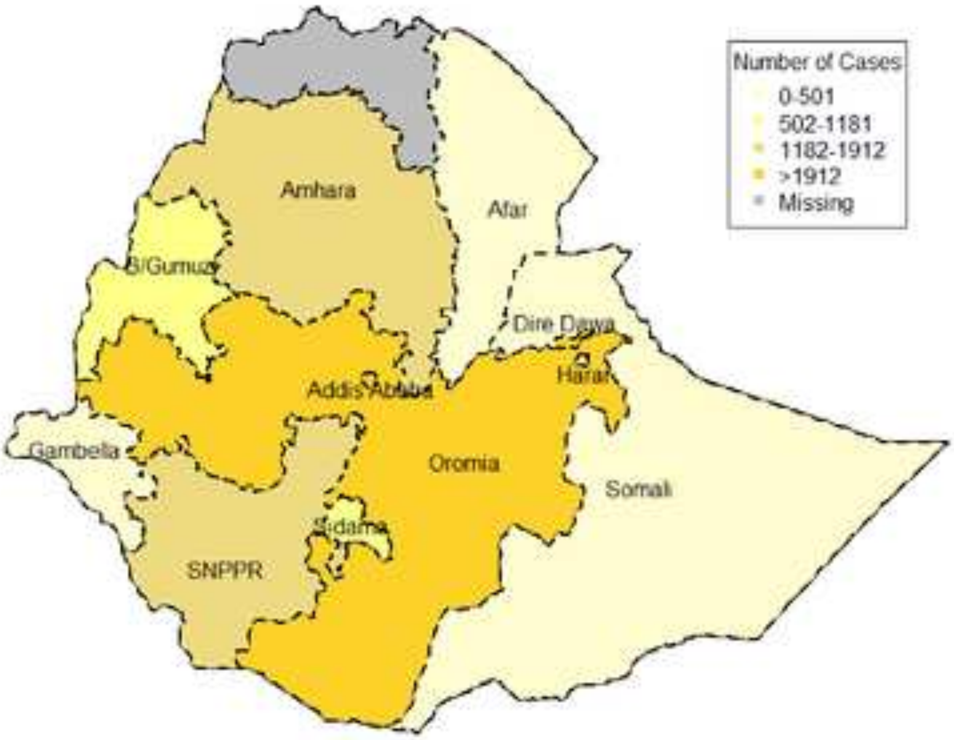

Figure 3

Click here to access/download;Figure;Figure 3.tiff

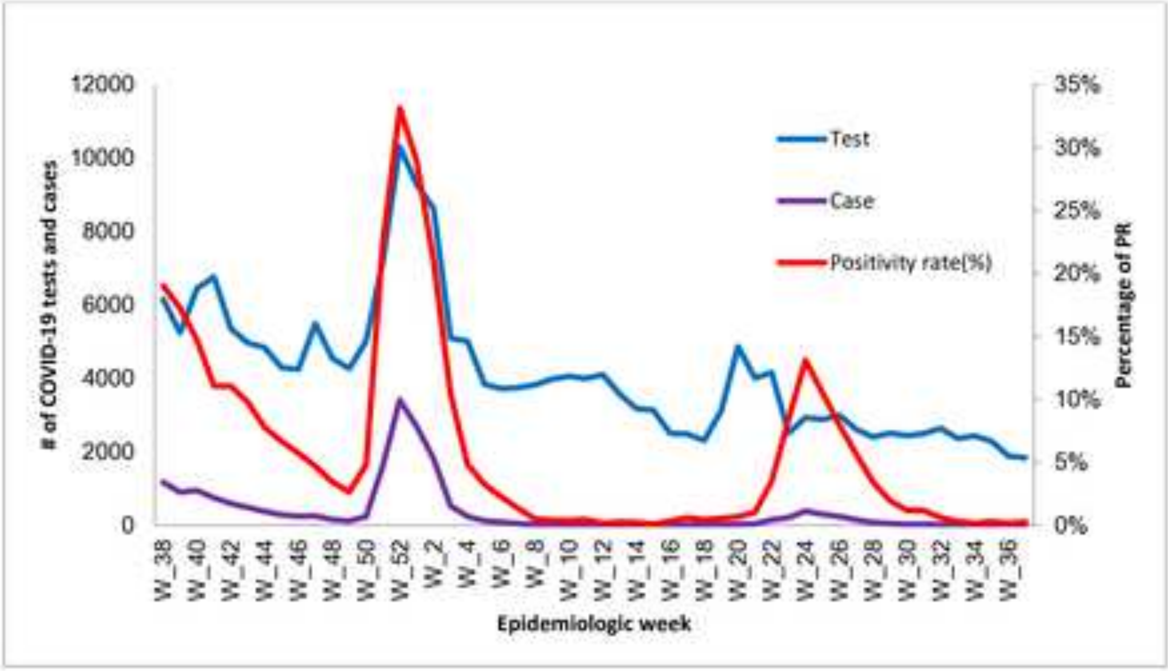

Figure 4

Click here to access/download;Figure;Figure 4.tiff

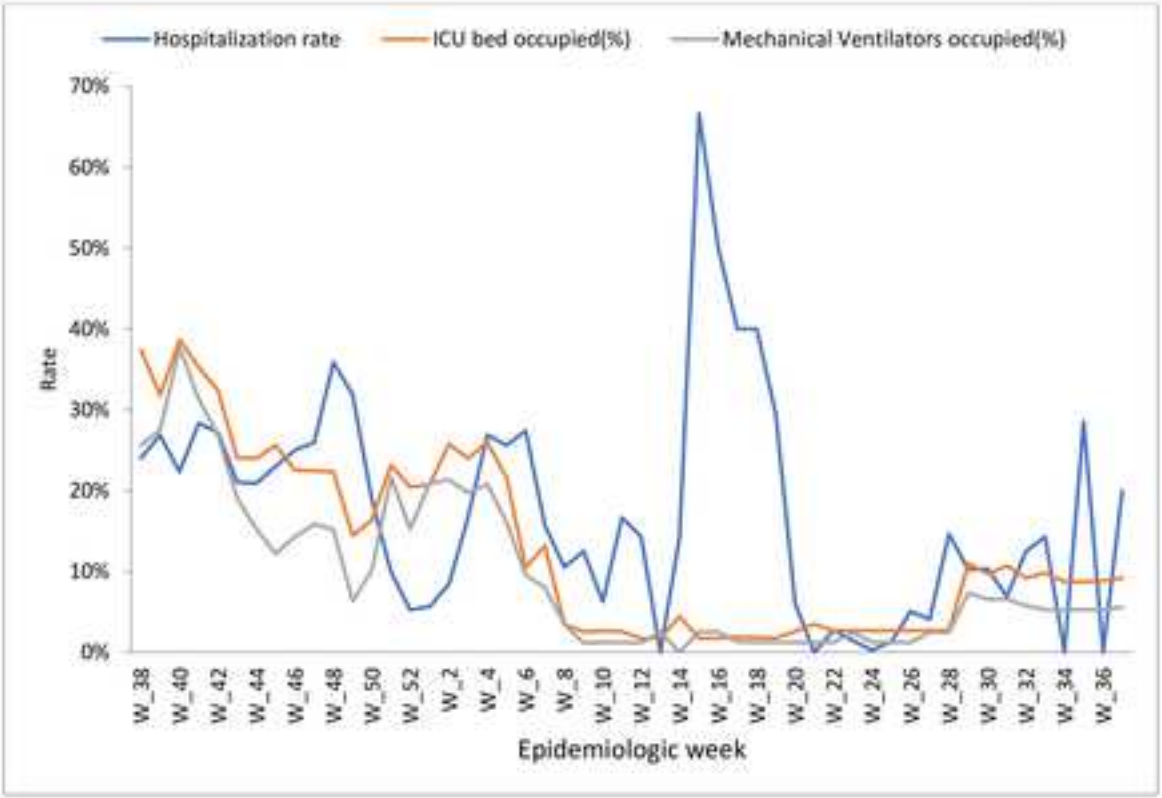

Figure 5

Click here to access/download;Figure;Figure 5.tiff

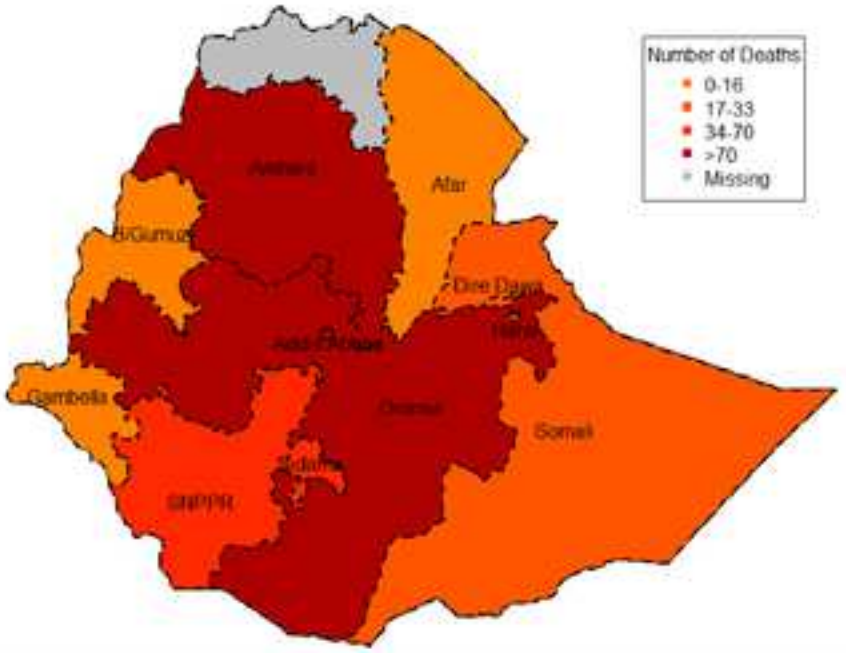

Supporting Information

Click here to access/download
Supporting Information
S1 Appendix.docx
