# Supplementary material for: Regional and temporal variations in COVID-19 cases and deaths in Ethiopia: Lessons learned from the COVID-19 enhanced surveillance and response
Source: PLOS Glob Public Health. 2024 May 23;4(5):e0003175. doi: 10.1371/journal.pgph.0003175 (PMC11115226; doi:10.1371/journal.pgph.0003175)
Supplement: Attachment — Submitted filename: Response to Reviewers.docx [file pgph.0003175.s005.docx]

Dear Editor and Reviewers,

Thank you very much for the insightful comments and suggested revisions, which have helped improve the quality of our manuscript. Please kindly find point-by-point responses to the comments as follows.

**Response to Editorial Inquiries**

1. Please include a complete copy of PLOS’ questionnaire on inclusivity in global research in your revised manuscript. Our policy for research in this area aims to improve transparency in the reporting of research performed outside of researchers’ own country or community. The policy applies to researchers who have travelled to a different country to conduct research, research with Indigenous populations or their lands, and research on cultural artefacts. The questionnaire can also be requested at the journal’s discretion for any other submissions, even if these conditions are not met.  Please find more information on the policy and a link to download a blank copy of the questionnaire here: <https://journals.plos.org/globalpublichealth/s/best-practices-in-research-reporting>. Please upload a completed version of your questionnaire as Supporting Information when you resubmit your manuscript.

**Response: We now uploaded a completed version of the inclusivity in global research questionnaire as Supporting Information.**

2. We have noticed that you have uploaded Supporting Information files, but you have not included a list of legends. Please add a full list of legends for your Supporting Information files after the references list.

**Response: We now included the list of legends.**

3. In the online submission form, you indicated that "All aggregated COVID-19 surveillance and response data used in this study will be made available in the EPHI data repository after publication. Access to data will be provided following the investigators’ approval of a proposal. Request for the data should be directed to the corresponding author. People requesting data will need to sign a data access agreement that will be forwarded upon request and the database will be transferred electronically". All PLOS journals now require all data underlying the findings described in their manuscript to be freely available to other researchers, either 1. In a public repository, 2. Within the manuscript itself, or 3. Uploaded as supplementary information.

**Response: Thank you. We now updated the data availability statement as “****All data underlying the findings described in the manuscript are available within the manuscript”.**

4. Some material included in your submission may be copyrighted. According to PLOS’s copyright policy, authors who use figures or other material (e.g., graphics, clipart, maps) from another author or copyright holder must demonstrate or obtain permission to publish this material under the Creative Commons Attribution 4.0 International (CC BY 4.0) License used by PLOS journals. Please closely review the details of PLOS’s copyright requirements here: PLOS Licenses and Copyright. If you need to request permissions from a copyright holder, you may use PLOS's Copyright Content Permission form. Please respond directly to this email or email the journal office and provide any known details concerning your material's license terms and permissions required for reuse, even if you have not yet obtained copyright permissions or are unsure of your material's copyright compatibility. Potential Copyright Issues: Figs 2 & 5: please (a) provide a direct link to the base layer of the map (i.e., the country or region border shape) and ensure this is also included in the figure legend; and (b) provide a link to the terms of use / license information for the base layer image or shapefile. We cannot publish proprietary or copyrighted maps (e.g. Google Maps, Mapquest) and the terms of use for your map base layer must be compatible with our CC-BY 4.0 license. Note: if you created the map in a software program like R or ArcGIS, please locate and indicate the source of the base map shapefile onto which data has been plotted.

**Response:** **We used R software to create the maps using the** **base map shape file from** [**https://data.humdata.org/dataset/cod-ab-eth**](https://data.humdata.org/dataset/cod-ab-eth)**. The use of the shape file is licensed under a Creative Commons Attribution 4.0 International license (**[**https://data.humdata.org/dataset/cod-ab-eth**](https://data.humdata.org/dataset/cod-ab-eth)**).**

**Response to Reviewer 1**

Reviewer 1: The authors did a good work in delineating the enhanced surveillance of COVID-19 and lessons learned in Ethiopia. Nevertheless, there are some areas in the manuscript need to be strengthened, including the main tables and figures. Please refer to my comments and their corresponding highlighted areas in the attachment.

Regarding statistical analysis, the authors need to consider adding 95% confidence intervals to the tables in main text to indicate uncertainties in the proportion estimates.

**Response: Thank you very much for the insightful comments and suggestions provided. We have incorporated all the suggestions you provided.**

R1: Abstract:

- It seems no geographic restriction was described in the conclusions.

**Response: Thank you. We now include Ethiopia in the conclusion.**

R1: Introduction

- Implemented where?

**Response: Thank you. We now include Ethiopia to indicate the implementation place**

- Write ICAP in its full name

**Response: Thank you. The full name, International Center for AIDS Program (ICAP), is provided now.**

- Given the limitations in the Discussion regarding the generalizability of the data/results in this study, it may be more defensible to say “is based on surveillance data at the national scale” here.

**Response: Thank you. Considering the limitations we have in the study, we agree to omit that.**

**R1:** Methods and Materials

- It seems this is not comparable to the other institutions at the primary healthcare level. Can the author clarify this?

**Response: In Ethiopia, a district (a local name “woreda”) is included in the primary healthcare level. A district (woreda) is the second lowest level of the administration hierarchy in Ethiopia, next to Kebele.**

- It seems “surveiling” would be more appropriate here.

**Response: Thank you. Revised accordingly.**

- It seems here can be simplified as “based on the”.

Response: **Thank you. Revised accordingly.**

- The COVID-19 pandemic started in late 2019 and spanned beyond the end of the study’s data collection period. Seeminly, the whole 2020 and a large portion of 2021 were missed in the data collection. Can the authors provide an justification for why the data prior to October 2021 and after October 2022 were not available?

**Response: Our collaborative project (CARES project) was launched in June 2021 and the well-established, monitored, quality-oriented surveillance data collection was started after the project was launched.**

- Can the authors clarify who provided the mentoring and supervision?

**Response: Thank you. It is revised as …a team of experts and researchers from the collaborating entities (EPHI, ICAP and GOHi) provided standardized training on COVID-19 surveillance…**

- Can the authors articulate who provided the feedbacks. For sentences like this, an active voice could be better than passive voice, as the sentence’s subject is spelled out.

**Response: Thank you. We revised the sentence as suggested.**

- Please provide its full form at first appearance.

**Response: Thank you. We spelled out DHIS2 as the District Health Information System.**

- Given Tables in main text are usually for results and the calculations metrics in this table are relatively self-explanatory. The authors may consider relocating this table to supplementary materials.

**Response: Thank you. We feel that Table 1 is important in the main text to provide optimal information on the measurement of variables for readers.**

R1: Results

- Please consider rephrase this sentence to - “In the study population (n=215,024) from 64 health facilities, consisted of COVID-19 suspected cases, contacts, and  travelers, 88.9% was tested by Antigen Detection Rapid Diagnostic Test (Ag RDT) and 11.1% was tested by Real-Time Polymerase Chain Reaction (RT-PCR)”.

**Response: Thank you. We now revise as suggested.**

- It seems this content duplicates the highlighted in the former paragraph. Please consider merging the former paragraph with this paragraph as the content in the former fits the heading here.

**Response: Thank you. We merged the two sentences now.**

- Other than showing this Map depicting the distribution of case count. Can the authors present a figure showing prevalence in each region? Without adjusting for population size in the count data, sometimes it is hard to geographically compare the relative burdens across regions. A potential option would be to visualize the prevalence estimates in Table 2 in a map. Also, for Figures 2 and 5, the authors need to add a scale bar and north arrow to each figure to strengthen interpretability.

**Response: Thank you. We now revised the map as suggested using the proportion rather than the absolute counts.**

- Can the author provide 95% confidence intervals for the proportions below to show their uncertainty? This comment applies to Tables 3, 4, and 5 as well.

**Response: Thank you. We now provided 95% CI for the proportions in Tables 2, 3, 4, and 5.**

- It seems the captions of all figures are not available in the manuscript for review. Can the authors add them for next iteration of review?

**Response: Thank you. We now provide captions of all figures.**

- Can the author provide the calendar date of this Epi-week to assist comparison with the data on the week of April 16, 22?

**Response: Thank you. We now include the relative calendar date for the Epi week 40, 2021.**

- Is this case faltality rate? Please provide its full form in first apprearance.

**Response: Yes. We provide its full form now.**

- When abbreviations like this appear in a table, the authors need to provide their full forms through a footnote.

**Response: Thank you. Footnotes included.**

- Can the authors explain why region-specific fatality rates are not available?

**Response: Thank you. We included that all the deaths recorded were from Addis Ababa.**

- Can the authors clarify how this portion of the contacts was selected for testing?

**Responses: We did not select the contacts to be tested. However, we traced contacts and able to test all willing contacts of the index cases.**

- Please consider providing the 95% CI as what have been done previously.

**Response: Thank you. We include the 95% CI for the estimate now.**

- Is the low prevalence in the two regions than Addis Ababa because more people were tested due to border crossing but had a lower positive rate? Or could the higher prevalence in Addis Ababa was aasociated with more person-to-person contacts due to a higher population density? Or could the variation due to uncertainties in these prevalence estimates (which may be indicated by the 95% confidence intervals)? Please elucidate these possible explanations in main text.

**Response: As you rightly stated, the higher prevalence in Addis Ababa is related to the high population density and more person-to-person contacts in the city than the regions.**

- It seems “generalizability” would be more appropriate here. Another factors which may impair the study’s generalizability to the general population in Ethiopia is selection bias - such bias could be caused by the fact that the study population was selected from facilities with high patient load, which may explain why some estimates like CFR from this study is higher. The authors need to discuss this point in limitation.

**Response: Thank you. We added “Further, the surveillance system may not adequately capture COVID-19 cases with an asymptomatic or mild clinical course at the community level “ to indicate the potential of the selection bias as one of the limitations of the study.**

**Response to Reviewer 2:**

The paper discusses the regional and temporal variations in COVID-19 cases and deaths in Ethiopia, based on data captured between October 2021 and September 2022. The study analyzed 215,024 individuals' data and found 18,964 COVID-19 cases and 534 deaths. The positivity rate ranged from 1% to 15% across different regions, and three waves of outbreaks were detected during the analysis period. The study also highlighted disparities in the COVID-19 surveillance and response system, emphasizing the need for concerted efforts to address these variations.

1. This paper has worked on data collection categorization and the authors have done a good job in this regard. However, the overall analytical approach of this study is not innovative and simplistic enough and the research conducted is mainly limited to the previous period. I think that research on such a sensitive topic should be more updated in terms of time scales of real-world scenarios to produce some new and meaningful results. I believe that more updated analytical methods could be used to explore some of the latest evolutions and contributions to the recent past.

**Response: Thank you for the comment. This manuscript is based on the surveillance data captured through a collaborative project and employed descriptive analysis. The nature of the data might not allow us to make further analytical or modelling analyses. However, for the sake of showing regional variations, we have included a 95% CI for the estimates.**

2. Provide more details on the research methods and data collection process. Explain the time frame for data collection, sample selection methods, and specific steps taken in data analysis to enhance the credibility and reproducibility of the study.

**Response: We implemented COVID-19 surveillance in 64 healthcare facilities in Ethiopia. The 64 facilities were selected based on high case-load reporting. We provided the following details in the manuscript:**

The healthcare facilities (health centers and hospitals) were responsible for surveilling COVID-19 cases and daily reporting to higher administrative levels. Our descriptive, cross-sectional study was conducted based on the COVID-19 surveillance and response data captured from 64 of these selected healthcare facilities between October 01, 2021 and September 30, 2022. Among the selected healthcare facilities, 31 were primary level, 21 were secondary level, and 12 were tertiary level healthcare facilities. Selection of facilities was systematically based on reported patient load (high-load facilities were selected), availability of COVID-19 testing service, and access to electricity. The number of healthcare facilities included was proportionally allocated to the regional states of Ethiopia to ensure representation of all geographical areas of the country. However, due to the conflict in Northern Ethiopia (Tigray region), we were unable to include that region in this study. The selected health facilities were supported by the CARES Act project through provision of designated public health surveillance officers responsible for carrying out COVID-19 surveillance activities to capture data and share reports to the next level in the health system.

To ensure the quality of surveillance data and minimize variability, a team of experts and researchers from the collaborating entities (EPHI, ICAP and GOHi) provided standardized training on COVID-19 surveillance to the public health surveillance officers. The team has also provided continuous mentorship and supportive supervision of officers at reporting facilities regularly (every two months). The officers at regional and central levels generated surveillance data completeness and timeliness reports and provided feedback continuously to the reporting facilities.

Surveillance data were captured in healthcare facilities using standardized COVID-19 surveillance tools and case definitions (S1 Appendix) adapted from the national COVID-19 surveillance guideline and the WHO COVID-19 surveillance interim guideline [16,17]. The deployed public health surveillance officers (Master of Public Health degree holders) were responsible for capturing COVID-19 surveillance data from clients, registering cases in the COVID-19 surveillance logbooks, and reporting to higher levels through the District Health Information System (DHIS2) or via e-mailing of Excel spreadsheets. The COVID-19 surveillance tools captured data on suspect case identification and testing status, number of confirmed cases, COVID-19 testing modality, number of admissions and deaths, contacts identified and tested, number of confirmed HCW cases and deaths, number of clusters/outbreaks investigated, and responses provided. Cluster investigation was conducted following WHO standard procedures (S1 Appendix) [18].

3. Clearly explain the trends in COVID-19 cases and deaths in different regions and periods in the discussion section. Provide more explanation and analysis of these trends, including their underlying causes and potential impacts.

**Response: Thank you. We included the disparities and reasons for the disparities in the discussion.**

4. Emphasize the importance of addressing disparities in the COVID-19 surveillance and response system highlighted in the study, and provide concrete recommendations for addressing these differences. This could include improving coordination and cooperation among monitoring and response systems in different regions to ensure more equitable and consistent response measures.

**Response: Thank you. We revised it as suggested.**

5. Discuss the importance of the study's results for COVID-19 response strategies and public health policies. Explore how these findings can be used to guide decision-making and resource allocation for more effective control and management of COVID-19 outbreaks.

**Response: Thank you. We emphasized the study’s result in supporting the COVID-19 response, particularly during the integration into the routine surveillance and response system.**
