# Supplementary material for: Regional and temporal variations in COVID-19 cases and deaths in Ethiopia: Lessons learned from the COVID-19 enhanced surveillance and response
Source: PLOS Glob Public Health. 2024 May 23;4(5):e0003175. doi: 10.1371/journal.pgph.0003175 (PMC11115226; doi:10.1371/journal.pgph.0003175)
Supplement: Attachment — Submitted filename: Editor comments.docx [file pgph.0003175.s006.docx]

Table 2: What % of samples tested positive by Ag-RDT and RT-PCR separately? Please include this.

Line 209: More than 200 clusters of outbreaks were identified. I did not find cluster definition in the method section. Suggest to add.

Any death was reported from clusters? Please mention.

Table 5: A large number of cases were detected from clusters. What types of test used to detect cluster cases? Suggest to add information.

Discussion: Add reference for the statement “COVID-19 has had a tremendous impact on the overall health, social, psychological, and economic wellbeing of the Ethiopian population.”

The first wave (October 2021) was may be attributed to the Delta variant. Suggest to include statistics from other study to compare or support your statement.

In the discussion section, authors mentioned three variants name for specific waves but nothing mention about the source of variant information in the method section. Clarify the issue and add information.

Add references for the statement “The overall positivity rate in our study was relatively lower than study reports from other countries.”

Add reference for “The COVID-19 CFR among all confirmed cases is higher in our study than the national CFR (2.8 compared to 2.0%) for the same period.

Any data about the vaccination among HCW? Vaccination impact has been discussed less in the discussion section. Suggest to add information about vaccination in the introduction section.
