# Supplementary material for: Regional and temporal variations in COVID-19 cases and deaths in Ethiopia: Lessons learned from the COVID-19 enhanced surveillance and response
Source: PLOS Glob Public Health. 2024 May 23;4(5):e0003175. doi: 10.1371/journal.pgph.0003175 (PMC11115226; doi:10.1371/journal.pgph.0003175)
Supplement: Attachment — Submitted filename: Response to Reviewers.docx [file pgph.0003175.s007.docx]

Dear Dr. Sukanta Chowdhury,

Thank you very much for the insightful comments and suggested revisions, which have helped improve the quality of our manuscript. Please kindly find point-by-point responses to the comments as follows.

1. Table 2: What % of samples tested positive by Ag-RDT and RT-PCR separately? Please include this.

**Response: Thank you. We now include the percentages of cases by testing modality.**

2. Line 209: More than 200 clusters of outbreaks were identified. I did not find cluster definition in the method section. Suggest to add.

**Response: We have included the definition of cluster in the Appendix 1 as follows:**

**COVID-19 Cluster:** A group of symptomatic individuals linked by time, geographic location and common exposures, containing at least one NAAT-confirmed case or at least two epidemiologically linked, symptomatic (meeting clinical criteria of suspect case definition A or B) persons with positive Ag-RDTs performed by a trained operator OR as a self-test (based on ≥97% specificity of test and desired >99.9% probability of at least one positive result being a true positive).

3. Any death was reported from clusters? Please mention.

**Response: We did not have a report of death from the clusters.**

4. Table 5: A large number of cases were detected from clusters. What types of test used to detect cluster cases? Suggest to add information.

**Response:** Both Ag-RDT and RT-PCR testing methods were used to detect cases from clusters.

5. Discussion: Add reference for the statement “COVID-19 has had a tremendous impact on the overall health, social, psychological, and economic wellbeing of the Ethiopian population.”

**Response: Thank you. We now add the reference.**

6. The first wave (October 2021) was may be attributed to the Delta variant. Suggest to include statistics from other study to compare or support your statement.

**Response: Thank you. We include now data on the increased hospitalization attributed to the delta variant from other studies.**

7. In the discussion section, authors mentioned three variants name for specific waves but nothing mention about the source of variant information in the method section. Clarify the issue and add information.

**Response: Thank you. The data regarding the variants of the SARS-COV-2 was obtained from the Ethiopian Public Health Institute COVID-19 Lab report and the WHO’s tracking system. We now have included this information in the methods section.**

8. Add references for the statement “The overall positivity rate in our study was relatively lower than study reports from other countries.”

**Response: Thank you. We now add the reference.**

9. Add reference for “The COVID-19 CFR among all confirmed cases is higher in our study than the national CFR (2.8 compared to 2.0%) for the same period.

**Response: Thank you. We now add the reference**

10. Any data about the vaccination among HCW? Vaccination impact has been discussed less in the discussion section. Suggest to add information about vaccination in the introduction section.

**Response: Thank you. We added some information about COVID-19 vaccination both in the Introduction and Discussion sections.**
